# Editorial: Objective and measurable predictors of violence risk and outcome among forensic patients with psychosis

**DOI:** 10.3389/fpsyt.2024.1359586

**Published:** 2024-01-19

**Authors:** Piyal Sen, Veena Kumari

**Affiliations:** ^1^Department of Life Sciences, College of Health, Medicine, and Life Sciences, Brunel University London, Uxbridge, United Kingdom; ^2^Department of Forensic Psychiatry, Elysium Healthcare, Milton Keynes, United Kingdom

**Keywords:** mental health, schizophraenia, EEG, violence, forensic - psychiatric practice

Forensic mental health services are tasked with predicting the risk of future violence for patients, to perform the very important societal function of keeping the public safe. The largest proportion of patients within forensic mental health services suffer from psychosis. Thus, it is crucially important that there are objective and measurable predictors of violence risk for this group. Forensic mental health services tend to be the most expensive services within health systems with the longest lengths of stay. Objective or numerical parameters of risk would help to introduce greater consistency into the risk assessment process, which would not only contribute to better public safety and reduce the risk of reputational damage to the service, but also help forensic clinicians to better defend their practice. This Research Topic thus focused on the objective and measurable predictors of violence risk with potential for translation into the clinical practice of risk assessment in forensic psychiatry. On this topic, we highlight four excellent studies as follows.

First, Guo et al. presented a systematic review and meta-analysis of the prevalence of different types of violence toward others among people with schizophrenia in China and their influencing factors. They categorized violence-to-others into four types: (i) the reviews of official criminal or psychiatric records, (ii) less serious forms of violence, (iii) acts which caused demonstrable harm to victims, and (iv) homicide. The findings showed a different prevalence for these violence types, with almost 1 in 4 from reviews of official records and less serious forms of violence. The findings also demonstrated how regional factors like being inland compared to coastal areas or being inpatients were important for predicting violence in China, although this was complicated by the fact that there were better detection rates of violence in areas with high levels of violence, thus contributing to better prevalence estimates. Age (< 40 years) was another predictor of violence. The authors, justifiably, concluded that prediction of violence in psychosis was complex as it depended on multiple factors which would need to be combined to improve accuracy of violence prediction.

The second study by Lin et al. in our series was also from China. This study provided promising evidence for an association between electroencephalogram (EEG)-based measures and violence in schizophrenia by comparing 43 violent patients with 51 non-violent patients. The findings revealed an abnormal pattern of EEG microstates, with the violent group exhibiting increased duration, occurrence and coverage of microstate class A and decreased occurrence of microstate class B relative to the non-violent group. The violent group also had lower probabilities of transitions from ‘B to C' and from ‘C to B', relative to the non-violent group. Microstates have been regarded by some researchers as representing the basic building blocks of consciousness, cognition and thought. Notably, this is the first study to explore the pattern of EEG microstates, comparing violent and non-violent patients with schizophrenia. The finding that violent patients with schizophrenia had an increased duration, coverage and incidence of microstate class A and a reduced occurrence of microstate class B, as well as lower transitional probability from “C to B” and “B to C” is thus a potentially exciting finding and needs replication in larger samples.

The third study by Wolf et al. specifically explored risk factors for violence among female forensic patients with schizophrenia spectrum disorders, with retrospective follow-up of patients discharged from forensic services. The findings showed that violence during the index offense was related to psychotic symptoms, while inpatient violence was associated with affective and behavioral instability, violent ideation/intermittent psychotic symptoms, and non-responsiveness to treatment. Furthermore, violent recidivism was related to non-compliance with treatment, cognitive instability, lack of insight, childhood antisocial behavior and poverty. This study thus highlights how different types of violence, namely violent index offense, inpatient violence and violent recidivism are predicted by different factors. This study highlighted another important factor in showing that the prediction of violence in women might not be that different to men as the additional assessment of the female addition manual (FAM) tools did not result in any improvement in predictive accuracy.

Lastly, Kruckl et al. examined whether there was any difference in outcomes between wards with an open-door or closed-door policy in a study carried out in Basel, Switzerland. Their findings showed, rather unsurprisingly, that there were more coercive measures like seclusion and involuntary medication in the wards which operate a closed-door policy as opposed to open-door policy. This was the case even in patients with previous experience of closed-door policies, thus concluding that clinical strategies to keep patients on the verge of coercion in open-door settings might have the potential to reduce overall incidents of coercion for in-patients. However, this study was handicapped by not following a properly randomized model and thus it is highly likely that patients who required more coercive strategies to manage them were moved from open wards to wards with a closed-door policy, thus potentially biasing the study findings. What this study highlights, however, is the importance of developing good predictors of violence risk which would improve risk management for early intervention to help reduce the need for coercive measures which have important human rights implications. With the incorporation of the United Nations Convention on the Rights of Person with Disabilities (UNCRPD) recommendations into mental healthcare, there is a real focus now on reducing coercive practices, and objective risk predictors of violence will assist with that.

To conclude, prediction of the risk of violence in schizophrenia is complex. It is hard to develop objective tools as the method of prediction might vary depending on various patient-specific and other broader factors, including socio-economic conditions. Nonetheless, the encouraging finding of EEG microstates as a potentially useful biological tool to predict violence in psychosis needs to be explored further. Furthermore, the risk predictor tools for women might not be that dissimilar to those used for men. The search for objective and measurable predictors of violence risk and outcome in psychosis thus needs to continue, while fully recognizing some of the caveats.

## Author contributions

PS: Conceptualization, Writing—original draft. VK: Conceptualization, Writing—review & editing.

